# Development of a clinical-radiological nomogram for predicting severe postoperative peritumoral brain edema following intracranial meningioma resection

**DOI:** 10.3389/fneur.2024.1478213

**Published:** 2025-01-16

**Authors:** Chen Bo, Geng Ao, Lu Siyuan, Wu Ting, Wang Dianjun, Zhao Nan, Shan Xiuhong, Deng Yan, Sun Eryi

**Affiliations:** ^1^Department of Neurosurgery, Affiliated People’s Hospital of Jiangsu University, Zhenjiang, China; ^2^Department of Radiology, Affiliated People’s Hospital of Jiangsu University, Zhenjiang, China; ^3^Department of Radiology, Northern Jiangsu People’s Hospital Affiliated to Yangzhou University, Yangzhou, China; ^4^Department of Pathology, Affiliated People’s Hospital of Jiangsu University, Zhenjiang, China; ^5^Department of Medical Record, Affiliated People’s Hospital of Jiangsu University, Zhenjiang, China; ^6^Department of Anesthesiology, West China Hospital, Sichuan University, Chengdu, China

**Keywords:** nomogram, PTBE, machine learning, meningioma, radiological

## Abstract

**Objective:**

The goal of this study was to develop a nomogram that integrates clinical data to predict the likelihood of severe postoperative peritumoral brain edema (PTBE) following the surgical removal of intracranial meningioma.

**Method:**

We included 152 patients diagnosed with meningioma who were admitted to the Department of Neurosurgery at the Affiliated People’s Hospital of Jiangsu University between January 2016 and March 2023. Clinical characteristics were collected from the hospital’s medical record system. Factors associated with severe postoperative PTBE were identified through univariate and LASSO regression analyses of clinical, pathological, and radiological features. A multivariate logistic regression analysis was then performed incorporating all features. Based on these analyses, we developed five predictive models using R software: conventional logistic regression, XGBoost, random forest, support vector machine (SVM), and k-nearest neighbors (KNN). Model performance was evaluated by calculating the area under the receiver operating characteristic curve (AUC) and conducting decision curve analysis (DCA). The most optimal model was used to create a nomogram for visualization. The nomogram was validated using both a validation set and clinical impact curve analysis. Calibration curves assessed the accuracy of the clinical-radiomics nomogram in predicting outcomes, with Brier scores used as an indicator of concordance. DCA was employed to determine the clinical utility of the models by estimating net benefits at various threshold probabilities for both training and testing groups.

**Results:**

The study involved 151 patients, with a prevalence of severe postoperative PTBE at 35.1%. Univariate logistic regression identified four potential risk factors, and LASSO regression identified four significant risk factors associated with severe postoperative PTBE. Multivariate logistic regression revealed three independent predictors: preoperative edema index, tumor enhancement intensity on MRI, and the number of large blood vessels supplying the tumor. Among all models, the conventional logistic model showed the best performance, with AUCs of 0.897 (95% CI: 0.829–0.965) and DCA scores of 0.719 (95% CI: 0.563–0.876) for each cohort, respectively. We developed a nomogram based on this model to predict severe postoperative PTBE in both training and testing cohorts. Calibration curves and Hosmer-Lemeshow tests indicated excellent agreement between predicted probabilities and observed outcomes. The Brier scores were 10.7% (95% CI: 6.7–14.7) for the training group and 25% (95% CI: 15.2–34.8) for the testing group. DCA confirmed that the nomogram provided superior net benefit across various risk thresholds for predicting severe postoperative PTBE, with a threshold probability range from 0 to 81%.

**Conclusion:**

Utilizing conventional logistic regression within machine learning frameworks, we developed a robust prediction model. The clinical-radiological nomogram, based on conventional logistic regression, integrated clinical characteristics to enhance the prediction accuracy for severe PTBE in patients following intracranial meningioma resection. This nomogram showed promise in aiding clinicians to create personalized and optimal treatment plans by providing precise forecasts of severe PTBE.

## Introduction

Meningiomas account for approximately 40% of primary central nervous system tumors (1), with most displaying indolent growth and being histologically classified as benign. Complete resection of benign meningiomas (WHO Grade I) often results in a curative outcome (2). However, postoperative peritumoral brain edema (PTBE) is a common complication following meningioma resection (3). Given that patients often present with mild symptoms upon admission, the development of PTBE can have severe consequences and significantly affect patient outcomes. Thus, it is crucial to address this complication effectively.

Research on predicting severe postoperative PTBE after meningioma resection remains limited. Venous injury is known to cause venous edema or infarction, potentially leading to local or distant venous congestion and subsequent PTBE (4). Some studies have proposed that “normal perfusion pressure breakthrough “may contribute to postoperative PTBE in meningiomas (5). This phenomenon could arise from disruptions in blood pressure autoregulation caused by the tumor mass in the surrounding brain tissue. The sudden increase in local blood flow following the removal of a large, vascularized meningioma could result in PTBE and intracerebral hemorrhages (6). Despite these insights, the exact pathogenesis of PTBE in meningiomas remains unclear. Therefore, this study investigated four main theories related to PTBE: brain parenchyma compression, secretory-excretory mechanisms, venous compression, and hydrodynamic factors (7). Additionally, various factors such as tumor location, margin characteristics, size, histology, sex hormones, tumor grade, vessel supply pattern, radiological features (e.g., degree of enhancement), and histopathological features (e.g., histological classification and Ki-67 labeling index [LI]) (5, 8–10) have been examined for their correlation with postoperative PTBE.

Machine learning (ML), a branch of artificial intelligence (AI), has increasingly been used in medical data analysis (11). ML techniques have been successfully applied in various clinical settings, including intensive care units (ICUs) (12, 13). In some cases, ML-based predictive tools have outperformed traditional statistical models in forecasting severe postoperative PTBE following meningioma resection. This study aimed to develop an ML-driven predictive model using preoperative and intraoperative clinical characteristics to estimate the likelihood of postoperative PTBE. Additionally, a nomogram was developed to visualize this predictive model.

## Methods

### Study design and patients

**Figure 1:**
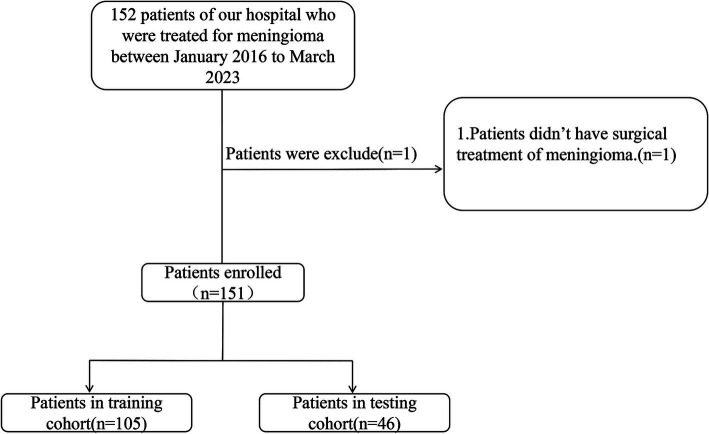
Recruitment pathway for eligible patients in this study.

This study enrolled the patients diagnosed with intracranial meningioma, who were admitted to the neurosurgery department at the Affiliated People’s Hospital of Jiangsu University between January 2016 and March 2023. Data were retrospectively collected from the clinical research data platform. After refining and extracting baseline information, these patients were randomly divided into training and validation groups in a 7:3 ratio (Figure 1). A random number generation algorithm was used to ensure the random allocation of patients between the training and validation groups. This retrospective study was approved by the Medical Ethics Committee of the Affiliated People’s Hospital of Jiangsu University (Approval No.K-20240115-W), in accordance with the Declaration of Helsinki. Informed consent was not required as all patient data were anonymized and de-identified prior to analysis.

### Clinical and surgical data

Comprehensive patient information, including age, gender, admission symptoms, diagnosis, and underlying conditions, was extracted from the medical record system. To ensure accuracy and consistency, domain experts meticulously verified each data point.

### Radiological data

Participants underwent standardized CT and MRI scans before and after meningioma resection using CUT 710 (United Imaging Healthcare Technology Co., Shanghai, China), Magnetom Skyra 3.0 T (Siemens, Erlangen, Germany), and UMR560 Imaging 1.5 T (United Imaging Healthcare Technology Co., Shanghai, China). The images were uploaded to a Picture Archiving and Communication System (PACS) for comprehensive analysis and long-term storage. Two independent neuroradiologists assessed the radiological images without access to clinical or pathological information. In cases of disagreement, a third neuroradiologist was consulted to reach a consensus on binary data decisions.

The edema index (EI) was calculated by dividing the combined volume of PTBE and tumor volume prior to surgery by the tumor volume observed on T2-weighted imaging (T2WI). Edema severity was classified as “none” (EI = 1.0), “moderate” (EI = 1.0–2.0), or “severe” (EI > 2.0). Postoperative PTBE was defined as an increase in volume on postoperative head CT scans, indicating a worsening of PTBE compared to preoperative levels, following meningioma resection, with or without hemorrhage (3, 14). Tumor volume was calculated using the spheroid formula (V = 4/3π × a/2 × b/2 × c/2) (10).

Additionally, the neuroradiologists evaluated various MR imaging techniques, including T1-weighted (T1WI), T2WI, fluid-attenuated inversion recovery, and contrast-enhanced T1-weighted (CE-T1WI) imaging. They also included magnetic resonance venography (MRV) and CT scans in their analysis. Based on these comprehensive evaluations, they extracted and documented the relevant radiological characteristics.

**Figure 2:**
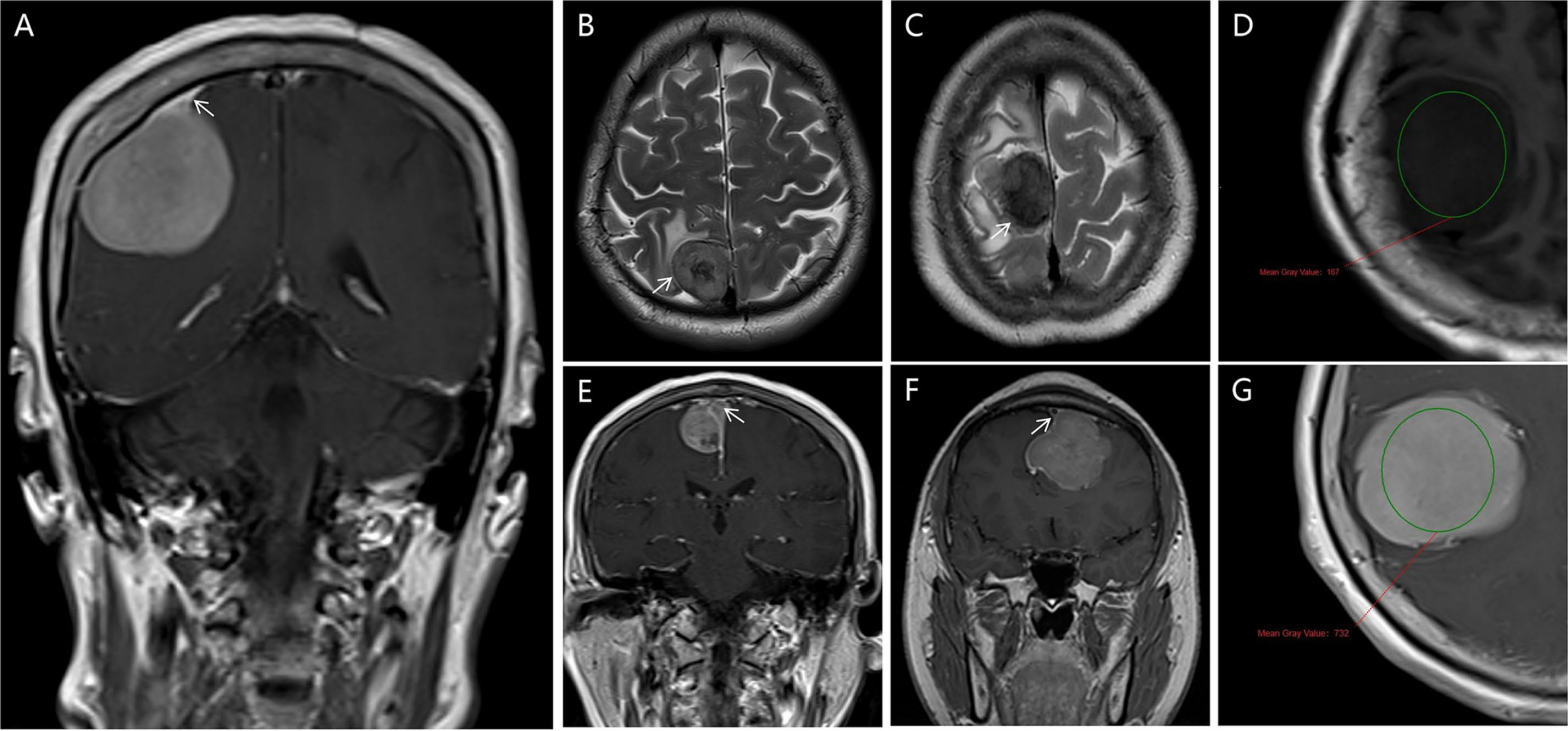
Radiological features are depicted in this study. **(A)** Dural Tail Sign in Weighted T1 MRI (white arrows pointed); **(B,C)** shows a CSF cleft in T1, and T2MRI image (white arrows pointed); **(D,G)** the degree of tumor enhancement was assessed by measuring the difference in grayscale values on T1WI before and after contrast enhancement of the solid tumor components (circles place); **(E,F)** the extent of venous sinus invasion was evaluated by analyzing the relationship between the tumor and the venous sinuses on MRV images (white arrows pointed).

(a) Tumor Location: Tumors were identified at various intracranial sites, including the convexity, falcine, and parasagittal regions, as well as in the anterior, middle, and posterior cranial fossae. Less common locations included the clivus, cerebellopontine angle, and foramen magnum. Tumors were also found in the parasellar region and involved the olfactory groove. Additionally, involvement of the petroclival area, tentorium, sphenoid wing, and tuberculum sellae was noted.
(b) Tumoral Calcification Assessment: In our investigation utilizing CT scans to detect calcifications, an attenuation value ≥80 HU (Hounsfield units) was used as a diagnostic criterion.
(c) Tumor Margin Classification: Tumor margins with lobulations or projections at the brain-tumor interface were classified as irregular. In contrast, margins that were smooth and lacked nodularity or indentation were categorized as smooth.
(d) Dural Tail Sign: The dural tail sign has been recognized as a common radiological feature in meningiomas, characterized by thickening and enhancement of the dura mater extending beyond the primary tumor mass (Figure 2A).
(e) Cerebrospinal fluid (CSF) Cleft: A CSF cleft is identified as a thin rim of CSF signal between the tumor and brain tissue. In this study, a CSF cleft was considered positive if visible in at least one imaging plane (axial, coronal, or sagittal) (Figures 2B,C).
(f) Degree of Tumor Enhancement: The degree of tumor enhancement was assessed by measuring the difference in grayscale values on T1WI before and after contrast enhancement of the solid tumor components. This method provides insights into the microcirculation and perfusion within the tumor tissue (Figures 2D,G).
(g) Assessment of Venous Sinus Invasion: The extent of venous sinus invasion was evaluated by analyzing the relationship between the tumor and the venous sinuses on MRV images. This analysis helps determine the degree of tumor infiltration into the venous structures (Figures 2E,F).
(h) Assessment of the Number of Large Blood Arteries Responsible for Tumor Blood Supply: In this study, “large blood arteries” supplying the meningioma were defined as vessels showing prominent contrast enhancement and significant caliber visible on axial, coronal, and sagittal CE-T1WI images. These arteries were identified through a comprehensive analysis of the aforementioned MRI sequences. All vessels suspected to be feeding the tumor were counted. The enumeration of these vessels was further verified by neurosurgeons during surgery to ensure accuracy, particularly for vessels that could not be clearly identified as arteries or veins in preoperative imaging (51).

### Histopathology and immunohistochemistry

Following the surgical removal of tumors, tissue samples were embedded in paraffin and prepared for histological examination using hematoxylin and eosin staining, in accordance with the WHO classification criteria for meningiomas (2007/2016). The histopathological analysis included the assessment of histological subtypes and grading based on WHO guidelines, as well as Ki-67 expression levels. Meningiomas were classified into common subtypes (meningothelial, transitional, fibrous, and psammomatous) and uncommon subtypes (angiomatous, microcystic, secretory, lymphoplasmacyte-rich, and metaplastic). Ki-67 immunohistochemistry staining was performed using a DM6000B microscope (Leica, Wetzlar, Germany) at ×400 magnification. The percentage of positively stained cells (Ki-67 LI [%]) was determined by analyzing six random fields from each slide with Image-Pro Plus 5.0 software (Media Cybernetics, Rockville, Maryland), calculating the ratio of positively stained cells to total cells.

### Risk factor selection and nomogram construction

Clinical parameters were initially analyzed using univariate logistic regression. Subsequently, all attributes were included in LASSO regression analysis, with the penalty parameter adjusted through tenfold cross-validation. Significant risk factors for severe postoperative PTBE identified by both LASSO and univariate analyses were then examined using multivariate logistic regression. These factors were combined using multivariate logistic regression along with ML algorithms, including extreme gradient boosting (XGBoost), random forest, support vector machine (SVM), and k-nearest neighbor (KNN). A user-friendly nomogram was developed for healthcare professionals based on these analyses (15, 16).

### Statistical analysis

Statistical analyses were performed using SPSS 22.0 and R software (version 4.1). Continuous variables were reported as median (interquartile range) or mean ± standard deviation (SD), depending on the results of the Shapiro–Wilk test, while categorical data were presented as proportions. Differences between groups were assessed using the Chi-squared test, Fisher’s exact test, and Mann–Whitney U test. Logistic regression was utilized to identify risk factors associated with outcomes, considering variables with a *p*-value <0.05 in univariate analysis for inclusion in stepwise logistic regression models.

ML algorithms have been known for their exceptional performance and have demonstrated significantly superior results compared to traditional regression approaches when predicting outcomes from extensive datasets (17). We adopted a diverse range of ML algorithms, including extreme gradient boosting (XGBoost), logistic regression, random forest, SVM, and KNN, to model the data. For model training, 70% of samples were randomly selected, with the remaining 30% reserved for testing. To prevent overfitting, necessary adjustments were made during training, and 5-fold cross-validation was used to determine the best hyperparameters. Optimized models were then applied in R to predict the risk of PTBE. Model performance on test sets was evaluated by calculating the area under the receiver operating characteristic curve (AUC), sensitivity, specificity, and overall accuracy. A higher AUC indicated better classification performance. The optimal algorithm was used to construct the nomogram, with its discrimination assessed using the concordance statistic and calibration evaluated through calibration curves and the minimum Akaike’s information criterion (AIC) regression model formulation. The nomogram’s performance was further validated by calculating its AUC, comparing predicted incidences with observed outcomes, and using Brier scores to evaluate the performance of the calibration curve. Decision curve analysis (DCA) was performed to assess clinical utility across various probability thresholds. Statistical significance was defined as a *p*-value <0.05.

## Results

### Patient characteristics

A retrospective analysis was conducted on data from 152 patients. One patient declined surgery and was excluded from analysis, resulting in a final cohort of 151 patients. The cohort was randomly divided into training and testing groups in a 7:3 ratio. No statistically significant differences were observed between the training and testing groups for any variables (Table 1, *p* > 0.05). The overall incidence of severe postoperative PTBE was 35.1% (53/151), with rates of 31.4% (33/105) in the training group and 43.5% (20/46) in the testing group.

**Table 1:** Baseline characteristics of the enrolled patients in the training and testing cohorts.

	[ALL] *N* = 151	Test *N* = 46	Train *N* = 105	*p* overall
Gender, *n* (%)				0.034
Female	105 (69.54%)	38 (82.61%)	67 (63.81%)	
Male	46 (30.46%)	8 (17.39%)	38 (36.19%)	
Age (years), media[Q1;Q3]	63.00 [52.00;69.00]	62.50 [52.25;72.00]	64.00 [52.00;69.00]	0.442
Symptoms on admission				
Headache, *n* (%)				0.864
Negative	92 (60.93%)	29 (63.04%)	63 (60.00%)	
Positive	59 (39.07%)	17 (36.96%)	42 (40.00%)	
Dizziness, *n* (%)				0.537
Negative	99 (65.56%)	28 (60.87%)	71 (67.62%)	
Positive	52 (34.44%)	18 (39.13%)	34 (32.38%)	
Vomit, *n* (%)				1.000
Negative	138 (91.39%)	42 (91.30%)	96 (91.43%)	
Positive	13 (8.61%)	4 (8.70%)	9 (8.57%)	
Limb weakness, *n* (%)				1.000
Negative	120 (79.47%)	37 (80.43%)	83 (79.05%)	
Positive	31 (20.53%)	9 (19.57%)	22 (20.95%)	
Tumor found by medical examination, *n* (%)				0.586
Negative	147 (97.35%)	44 (95.65%)	103 (98.10%)	
Positive	4 (2.65%)	2 (4.35%)	2 (1.90%)	
Medical history				
Diabetes, *n* (%)				0.775
Negative	136 (90.07%)	41 (89.13%)	95 (90.48%)	
Positive	15 (9.93%)	5 (10.87%)	10 (9.52%)	
Hypertension, *n* (%)				0.888
Negative	85 (56.29%)	25 (54.35%)	60 (57.14%)	
Positive	66 (43.71%)	21 (45.65%)	45 (42.86%)	
Heart disease, *n* (%)				0.518
Negative	149 (98.68%)	45 (97.83%)	104 (99.05%)	
Positive	2 (1.32%)	1 (2.17%)	1 (0.95%)	
Brain trauma, *n* (%)				0.166
Negative	146 (96.69%)	43 (93.48%)	103 (98.10%)	
Positive	5 (3.31%)	3 (6.52%)	2 (1.90%)	
Epilepsy, *n* (%)				0.553
Negative	137 (90.73%)	43 (93.48%)	94 (89.52%)	
Positive	14 (9.27%)	3 (6.52%)	11 (10.48%)	
Number of large blood artery responsible for tumor blood supply, *n* (%)				0.734
0	8 (5.30%)	3 (6.52%)	5 (4.76%)	
1	109 (72.19%)	32 (69.57%)	77 (73.33%)	
2	24 (15.89%)	9 (19.57%)	15 (14.29%)	
3	10 (6.62%)	2 (4.35%)	8 (7.62%)	
Extracarotid artery (tumor blood supply), *n* (%)				0.244
Negative	60 (39.74%)	22 (47.83%)	38 (36.19%)	
Positive	91 (60.26%)	24 (52.17%)	67 (63.81%)	
Internal carotid artery (tumor blood supply), *n* (%)				0.553
Negative	102 (67.55%)	29 (63.04%)	73 (69.52%)	
Positive	49 (32.45%)	17 (36.96%)	32 (30.48%)	
Vertebral artery (tumor blood supply), *n* (%)				1.000
Negative	132 (87.42%)	40 (86.96%)	92 (87.62%)	
Positive	19 (12.58%)	6 (13.04%)	13 (12.38%)	
Drainage of venous disturbance, *n* (%)				0.429
Negative	110 (72.85%)	36 (78.26%)	74 (70.48%)	
Positive	41 (27.15%)	10 (21.74%)	31 (29.52%)	
Venous sinus disturbance, *n* (%)				0.577
Negative	112 (74.17%)	36 (78.26%)	76 (72.38%)	
Positive	39 (25.83%)	10 (21.74%)	29 (27.62%)	
The intensity of tumor enhancement on MRI, media[Q1;Q3]	447.00 [311.01;585.26]	450.88 [340.00;550.50]	425.90 [292.90;590.52]	0.527
Peritumoral Hypointensity on T2 Weighted MRI, *n* (%)				0.334
Negative	88 (58.28%)	30 (65.22%)	58 (55.24%)	
Positive	63 (41.72%)	16 (34.78%)	47 (44.76%)	
Meningoceal signs, *n* (%)				0.068
Negative	10 (6.62%)	6 (13.04%)	4 (3.81%)	
Positive	141 (93.38%)	40 (86.96%)	101 (96.19%)	
Operation time (hour), media[Q1;Q3]	5.00 [4.00;7.21]	5.00 [4.00;7.00]	5.00 [4.00;7.50]	0.857
Meningioma location				
Foramen magnum, *n* (%)				1.000
Negative	150 (99.34%)	46 (100.00%)	104 (99.05%)	
Positive	1 (0.66%)	0 (0.00%)	1 (0.95%)	
Anterior fossa base, *n* (%)				0.166
Negative	146 (96.69%)	43 (93.48%)	103 (98.10%)	
Positive	5 (3.31%)	3 (6.52%)	2 (1.90%)	
Fossae temporalis, *n* (%)				1.000
Negative	127 (84.11%)	39 (84.78%)	88 (83.81%)	
Positive	24 (15.89%)	7 (15.22%)	17 (16.19%)	
Occipital lobe, *n* (%)				0.311
Negative	140 (92.72%)	41 (89.13%)	99 (94.29%)	
Positive	11 (7.28%)	5 (10.87%)	6 (5.71%)	
Sphenoid ridge, *n* (%)				0.462
Negative	128 (84.77%)	37 (80.43%)	91 (86.67%)	
Positive	23 (15.23%)	9 (19.57%)	14 (13.33%)	
Petroclival, *n* (%)				1.000
Negative	146 (96.69%)	45 (97.83%)	101 (96.19%)	
Positive	5 (3.31%)	1 (2.17%)	4 (3.81%)	
Cavernous sinus, *n* (%)				0.127
Negative	137 (90.73%)	39 (84.78%)	98 (93.33%)	
Positive	14 (9.27%)	7 (15.22%)	7 (6.67%)	
Paracele, *n* (%)				1.000
Negative	149 (98.68%)	46 (100.00%)	103 (98.10%)	
Positive	2 (1.32%)	0 (0.00%)	2 (1.90%)	
Cerebellar peduncle, *n* (%)				0.775
Negative	136 (90.07%)	41 (89.13%)	95 (90.48%)	
Positive	15 (9.93%)	5 (10.87%)	10 (9.52%)	
Tentorium, *n* (%)				0.724
Negative	141 (93.38%)	44 (95.65%)	97 (92.38%)	
Positive	10 (6.62%)	2 (4.35%)	8 (7.62%)	
Falx cerebri, *n* (%)				0.436
Negative	144 (95.36%)	43 (93.48%)	101 (96.19%)	
Positive	7 (4.64%)	3 (6.52%)	4 (3.81%)	
Convexity, *n* (%)				0.124
Negative	76 (50.33%)	28 (60.87%)	48 (45.71%)	
Positive	75 (49.67%)	18 (39.13%)	57 (54.29%)	
Multiple meningiomas, *n* (%)				0.255
Negative	135 (89.40%)	39 (84.78%)	96 (91.43%)	
Positive	16 (10.60%)	7 (15.22%)	9 (8.57%)	
Venous not injury in surgery, *n* (%)	151 (100.00%)	46 (100.00%)	105 (100.00%)	
Venous decompensation, *n* (%)				1.000
Negative	145 (96.03%)	44 (95.65%)	101 (96.19%)	
Positive	6 (3.97%)	2 (4.35%)	4 (3.81%)	
Postoperative bleeding, *n* (%)				0.590
Negative	82 (54.30%)	27 (58.70%)	55 (52.38%)	
Positive	69 (45.70%)	19 (41.30%)	50 (47.62%)	
Preoperative midline displacement, *n* (%)				0.825
Negative	115 (76.16%)	34 (73.91%)	81 (77.14%)	
Positive	36 (23.84%)	12 (26.09%)	24 (22.86%)	
Preoperative use of mannitol, *n* (%)				0.872
Negative	95 (62.91%)	28 (60.87%)	67 (63.81%)	
Positive	56 (37.09%)	18 (39.13%)	38 (36.19%)	
Recurrence, *n* (%)				1.000
Negative	143 (94.70%)	44 (95.65%)	99 (94.29%)	
Positive	8 (5.30%)	2 (4.35%)	6 (5.71%)	
Meningeal tumor volume (mm^3^), media[Q1;Q3]	21528.00 [9439.50;39910.50]	21903.75 [8000.00;45371.25]	21528.00 [9900.00;38000.00]	0.811
Preoperative edema index, media[Q1;Q3]	1.10 [1.00;1.35]	1.10 [1.00;1.48]	1.00 [1.00;1.30]	0.775
Pathology				
Protruding nucleoli, *n* (%)				0.518
Negative	149 (98.68%)	45 (97.83%)	104 (99.05%)	
Positive	2 (1.32%)	1 (2.17%)	1 (0.95%)	
Classification of meningioma (WHO), *n* (%)				1.000
1	148 (98.01%)	45 (97.83%)	103 (98.10%)	
2	3 (1.99%)	1 (2.17%)	2 (1.90%)	
Meningoepithelial, *n* (%)				1.000
Negative	124 (82.12%)	38 (82.61%)	86 (81.90%)	
Positive	27 (17.88%)	8 (17.39%)	19 (18.10%)	
Angiomatous meningioma, *n* (%)				1.000
Negative	131 (86.75%)	40 (86.96%)	91 (86.67%)	
Positive	20 (13.25%)	6 (13.04%)	14 (13.33%)	
Transitional, *n* (%)				1.000
Negative	129 (85.43%)	39 (84.78%)	90 (85.71%)	
Positive	22 (14.57%)	7 (15.22%)	15 (14.29%)	
Fibrous, *n* (%)				0.838
Negative	95 (62.91%)	30 (65.22%)	65 (61.90%)	
Positive	56 (37.09%)	16 (34.78%)	40 (38.10%)	
Microcystic, *n* (%)				0.668
Negative	145 (96.03%)	45 (97.83%)	100 (95.24%)	
Positive	6 (3.97%)	1 (2.17%)	5 (4.76%)	
Psammomatous, *n* (%)				0.068
Negative	141 (93.38%)	40 (86.96%)	101 (96.19%)	
Positive	10 (6.62%)	6 (13.04%)	4 (3.81%)	
Mixed meningioma, *n* (%)				0.676
Negative	144 (95.36%)	45 (97.83%)	99 (94.29%)	
Positive	7 (4.64%)	1 (2.17%)	6 (5.71%)	
KI67(%), media[Q1;Q3]	2.00 [1.00;5.00]	2.00 [1.00;5.00]	2.00 [1.00;5.00]	0.323
Cystic meningioma, *n* (%)				1.000
Negative	143 (94.70%)	44 (95.65%)	99 (94.29%)	
Positive	8 (5.30%)	2 (4.35%)	6 (5.71%)	
Calcification, *n* (%)				0.668
Negative	145 (96.03%)	45 (97.83%)	100 (95.24%)	
Positive	6 (3.97%)	1 (2.17%)	5 (4.76%)	
EMA, *n* (%)				1.000
Negative	5 (3.31%)	1 (2.17%)	4 (3.81%)	
Positive	146 (96.69%)	45 (97.83%)	101 (96.19%)	
VIM *n* (%): Positive	151 (100.00%)	46 (100.00%)	105 (100.00%)	
S100, *n* (%)				1.000
Negative	139 (92.05%)	43 (93.48%)	96 (91.43%)	
Positive	12 (7.95%)	3 (6.52%)	9 (8.57%)	
GFAP, *n* (%)				1.000
Negative	150 (99.34%)	46 (100.00%)	104 (99.05%)	
Positive	1 (0.66%)	0 (0.00%)	1 (0.95%)	
P53, *n* (%)				0.919
Negative	129 (85.43%)	40 (86.96%)	89 (84.76%)	
Positive	22 (14.57%)	6 (13.04%)	16 (15.24%)	
PR, *n* (%)				0.549
Negative	29 (19.21%)	7 (15.22%)	22 (20.95%)	
Positive	122 (80.79%)	39 (84.78%)	83 (79.05%)	

### Selection of risk factors

**Figure 3:**
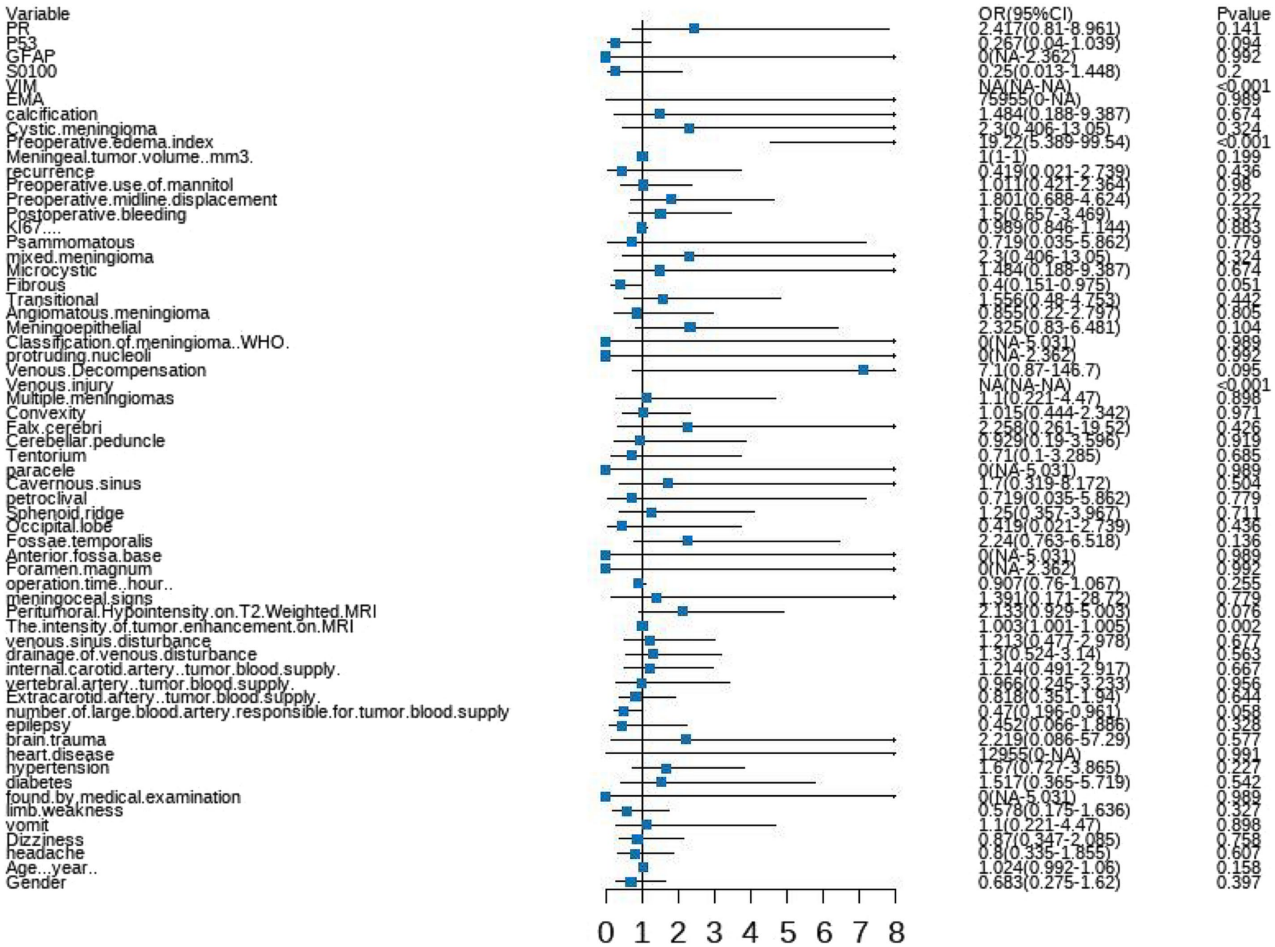
Forest plot illustrating the characteristics identified through univariate logistic regression analyses.

**Figure 4:**
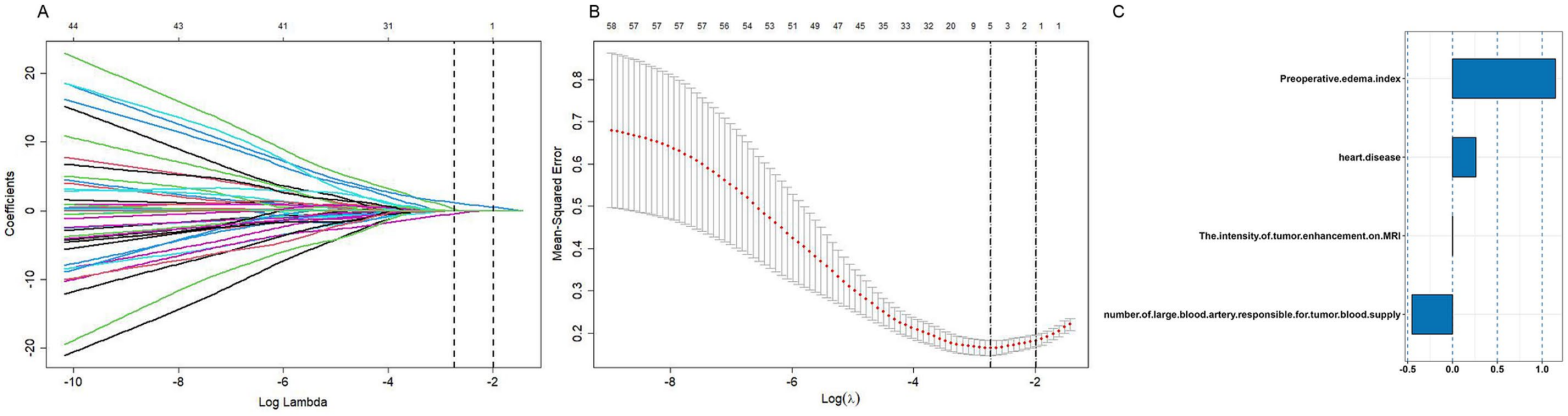
Parameter tuning for LASSO regression in the training cohort. **(A)** LASSO coefficient profiles for clinical features. **(B)** Optimal penalization coefficient (lambda) determined via 10-fold cross-validation. The lambda value corresponding to the minimum mean squared error for the training cohort is shown. **(C)** Positive characteristics identified by LASSO regression.

**Figure 5:**
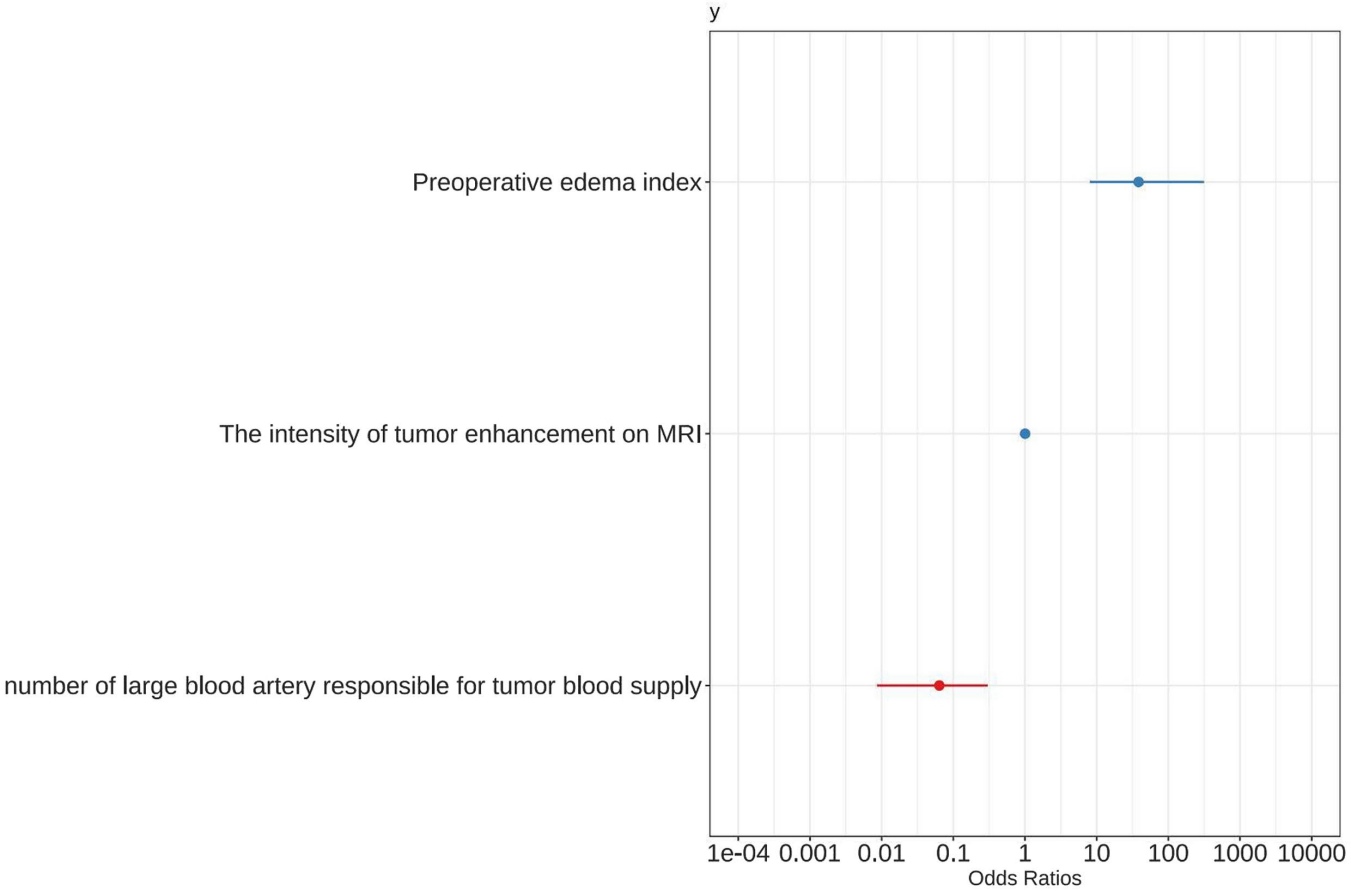
Forest plot showing positive characteristics identified through multivariate logistic regression analyses.

A forest plot was used to visually represent the various risk factors associated with severe postoperative PTBE following meningioma resection, based on univariate logistic regression analyses (Figure 3). Four risk factors with a *p*-value less than 0.05 were identified and further analyzed using LASSO regression to highlight the most significant risk factors linked to severe PTBE (Figure 4). Multivariate logistic regression analyses (Table 2) isolated three key determinants for severe PTBE after meningioma resection: preoperative EI, tumor enhancement intensity on MRI, and the number of large blood vessels supplying the tumor. These associations were also illustrated in a forest plot (Figure 5).

**Table 2:** Results of univariate and multivariate logistic regression analyses.

Univariate analysis				Multivariate analysis			
β coefficient	OR	95% CI	*p*-value	β coefficient	OR	95% CI	*p*-value
−0.381	0.683	0.683(0.275–1.62)	0.397				
0.024	1.024	1.024(0.992–1.06)	0.158				
−0.223	0.8	0.8(0.335–1.855)	0.607				
−0.14	0.87	0.87(0.347–2.085)	0.758				
0.095	1.1	1.1(0.221–4.47)	0.898				
−0.549	0.578	0.578(0.175–1.636)	0.327				
−14.814	0	0(NA–5.031)	0.989				
0.417	1.517	1.517(0.365–5.719)	0.542				
0.513	1.67	1.67(0.727–3.865)	0.227				
16.377	12955078.93	12,955(0-NA)	0.991				
0.797	2.219	2.219(0.086–57.29)	0.577				
−0.795	0.452	0.452(0.066–1.886)	0.328				
−0.755	0.47	0.47(0.196–0.961)	0.058	−2.752	0.064	0.063 (0.008–0.293)	0.002
−0.2	0.818	0.818(0.351–1.94)	0.644				
−0.035	0.966	0.966(0.245–3.233)	0.956				
0.194	1.214	1.214(0.491–2.917)	0.667				
0.262	1.3	1.3(0.524–3.14)	0.563				
0.193	1.213	1.213(0.477–2.978)	0.677				
0.003	1.003	1.003(1.001–1.005)	0.002	0.003	1.003	1.003 (1.000–1.006)	0.024
0.757	2.133	2.133(0.929–5.003)	0.076				
0.33	1.391	1.391(0.171–28.72)	0.779				
−0.098	0.907	0.907(0.76–1.067)	0.255				
−14.8	0	0(NA–2.362)	0.992				
−14.814	0	0(NA–5.031)	0.989				
0.806	2.24	2.24(0.763–6.518)	0.136				
−0.87	0.419	0.419(0.021–2.739)	0.436				
0.223	1.25	1.25(0.357–3.967)	0.711				
−0.33	0.719	0.719(0.035–5.862)	0.779				
0.531	1.7	1.7(0.319–8.172)	0.504				
−14.814	0	0(NA–5.031)	0.989				
−0.343	0.71	0.71(0.1–3.285)	0.685				
−0.074	0.929	0.929(0.19–3.596)	0.919				
0.815	2.258	2.258(0.261–19.52)	0.426				
0.015	1.015	1.015(0.444–2.342)	0.971				
0.095	1.1	1.1(0.221–4.47)	0.898				
−0.78		NA(NA–NA)	<0.01				
1.96	7.1	7.1(0.87–146.7)	0.095				
−14.8	0	0(NA–2.362)	0.992				
−14.814	0	0(NA–5.031)	0.989				
0.844	2.325	2.325(0.83–6.481)	0.104				
−0.156	0.855	0.855(0.22–2.797)	0.805				
0.442	1.556	1.556(0.48–4.753)	0.442				
−0.916	0.4	0.4(0.151–0.975)	0.051				
0.395	1.484	1.484(0.188–9.387)	0.674				
0.833	2.3	2.3(0.406–13.05)	0.324				
−0.33	0.719	0.719(0.035–5.862)	0.779				
−0.011	0.989	0.989(0.846–1.144)	0.883				
0.405	1.5	1.5(0.657–3.469)	0.337				
0.588	1.801	1.801(0.688–4.624)	0.222				
0.011	1.011	1.011(0.421–2.364)	0.98				
−0.87	0.419	0.419(0.021–2.739)	0.436				
0	1	1(1–1)	0.199				
2.956	19.22	19.22(5.389–99.54)	<0.01	3.648	38.402	38.40 (8.054–307.3)	<0.01
0.833	2.3	2.3(0.406–13.05)	0.324				
0.395	1.484	1.484(0.188–9.387)	0.674				
15.843	7595513.096	75,955(0-NA)	0.989				
−0.78		NA(NA–NA)	<0.01				
−1.386	0.25	0.25(0.013–1.448)	0.2				
−14.8	0	0(NA–2.362)	0.992				
−1.319	0.267	0.267(0.04–1.039)	0.094				
0.882	2.417	2.417(0.81–8.961)	0.141				

### Model construction and selection

**Figure 6:**
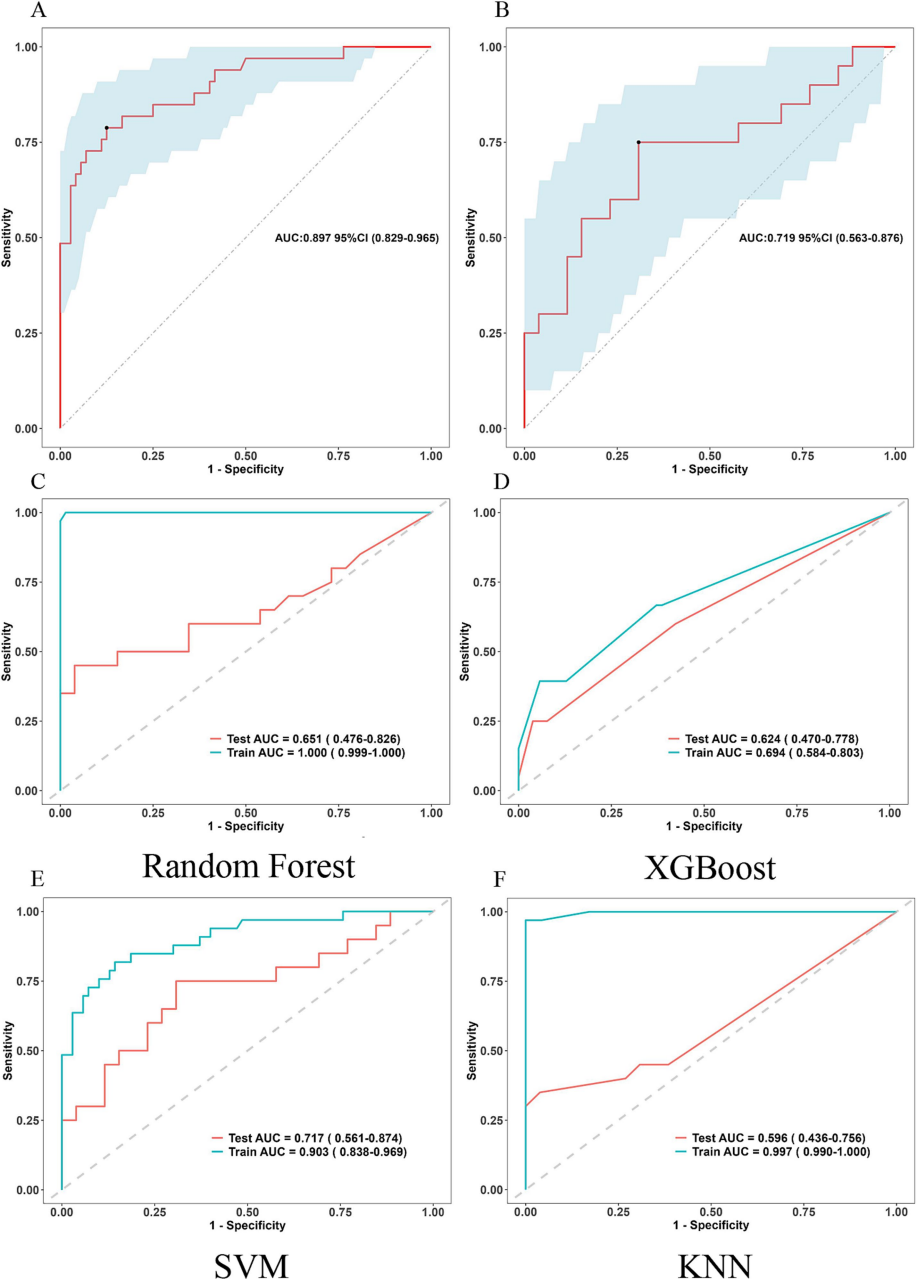
ROC curves for assessing the five ML classification techniques employed to predict postoperative PTBE status in meningioma patients. **(A,B)** Logistic regression Training set and Testing set. ROC: Receiver Operating Characteristic (bootstrap replicates = 500). **(C)** ROC curves for random forest, **(D)** ROC curves for XGBoost, **(E)** ROC curves for SVM, **(F)** ROC curves for KNN.

Five ML classification techniques were employed to predict postoperative PTBE status in meningioma patients: logistic regression, random forest, XGBoost, SVM, and KNN. ROC curves were generated and AUC values calculated for each model using both training and testing datasets (Figure 6). The logistic regression-based model demonstrated superior predictive capabilities in testing set, indicating its robustness.

### Model visualization and evaluation

**Figure 7:**
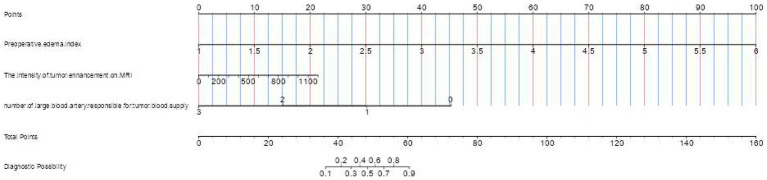
Nomogram based on multivariate logistic regression analysis for predicting the occurrence of chronic hydrocephalus in patients with aneurysmal subarachnoid hemorrhage.

The model developed in this study was visualized using a nomogram, a graphical tool designed to enhance its clinical utility (Figure 7). To estimate the probability of severe postoperative PTBE after meningioma resection for an individual, each factor in the nomogram was assigned a score ranging from 0 to 100 based on its regression coefficient related to severe PTBE. By drawing perpendicular lines from each factor’s axis to the points axis and summing these scores, we calculated a cumulative score. This total score was then mapped to the final score scale to estimate the likelihood of severe PTBE.

The nomogram’s predictive performance was evaluated using ROC analyses. The AUC values for the training and testing groups were 0.897 (95% CI: 0.829–0.965) and 0.719 (95% CI: 0.563–0.876), respectively (Figures 6A,B).

**Figure 8:**
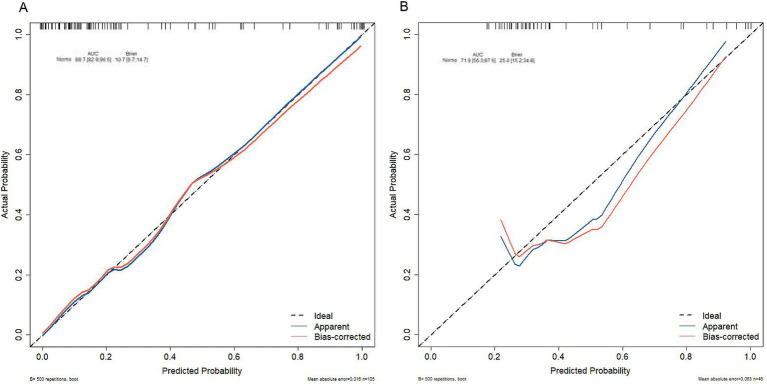
Calibration curve analysis of the nomogram in **(A)** the training set and **(B)** the testing set.

Brier scores were calculated to further assess the model’s accuracy. The scores were 10.7% (95% CI: 6.7–14.7) for the training group and 25% (95% CI: 15.2–34.8) for the testing group, reflecting a good concordance between the nomogram’s predictions and actual outcomes, as shown by the calibration curve (Figure 8).

**Figure 9:**
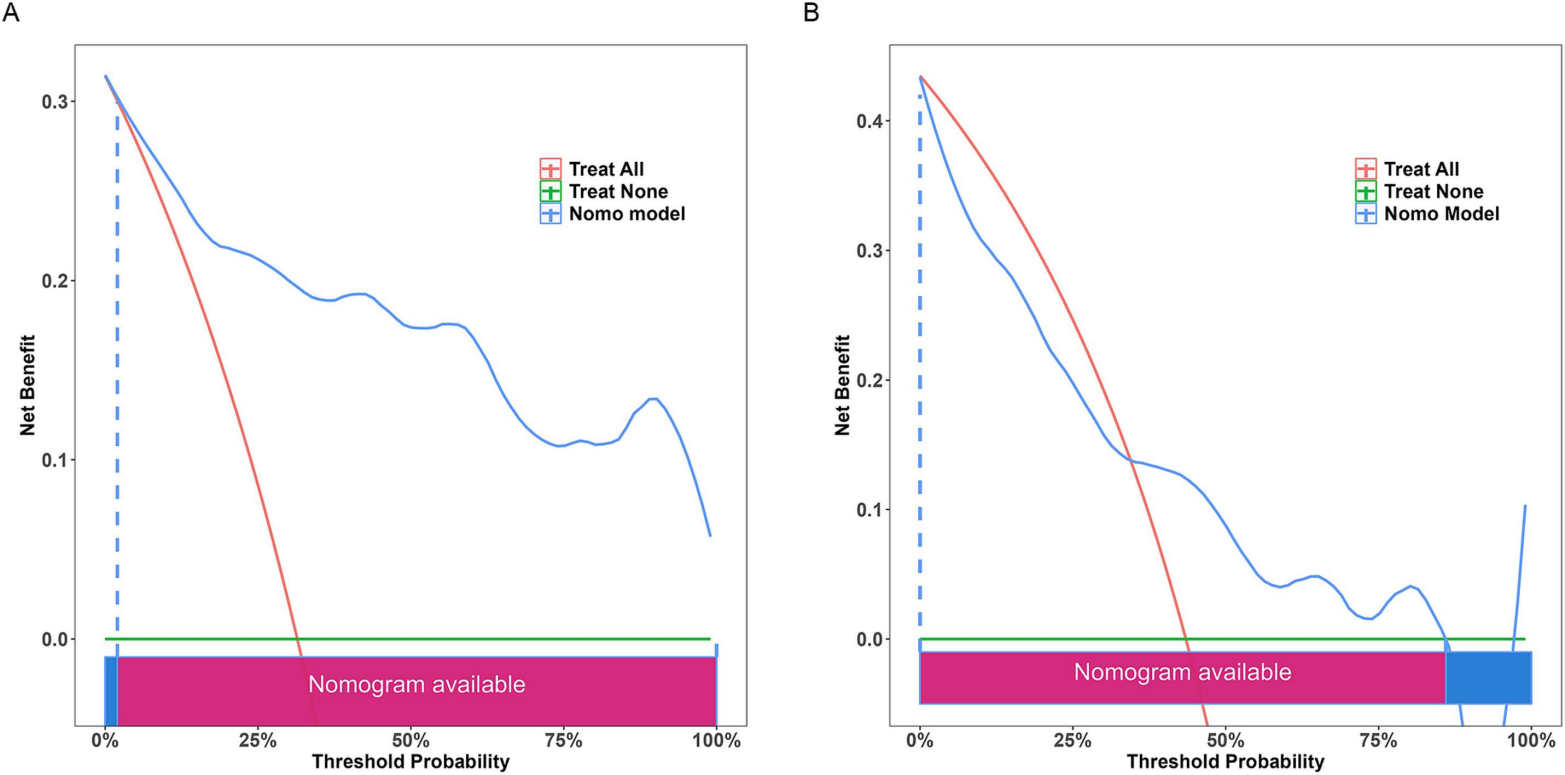
Decision curve analysis of the nomogram. **(A)** Training set; **(B)** testing set. The decision curve indicates that using this model to predict the occurrence of severe postoperative PTBE after meningioma resection is more effective when the threshold probability is between 0 and 80%.

The results of DCA (Figure 9) indicated that the nomogram provided significant predictive advantages across a range of threshold probabilities from 0 to 80%.

**Figure 10:**
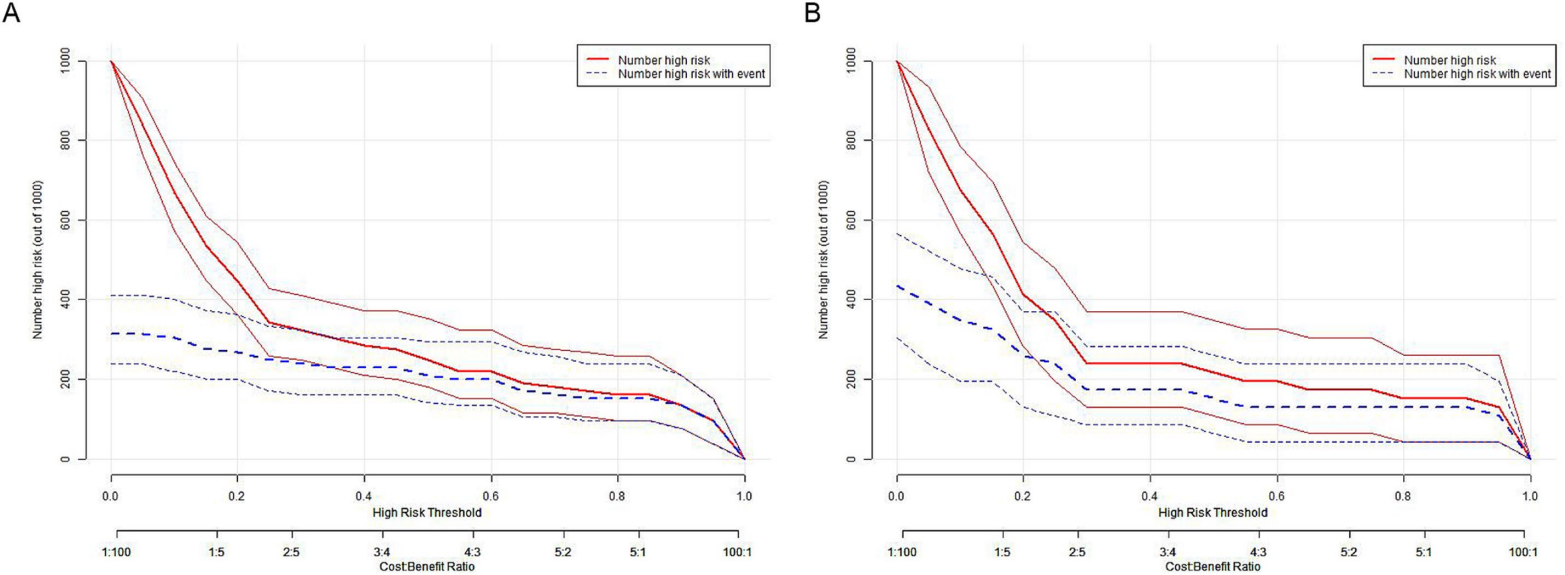
Clinical impact curve (CIC) analysis in **(A)** the training set and **(B)** the testing set.

Clinical impact curve (CIC) analysis (Figure 10) demonstrated that the nomogram offered a superior net benefit across a practical range of threshold probabilities, indicating its potential to improve patient outcomes substantially. These findings highlight the considerable predictive value of the logistic model used in this study.

## Discussion

This study successfully developed a clinical nomogram that provided a valuable tool for predicting severe postoperative PTBE following meningioma resection. The nomogram offered clinicians a solid basis for assessing individual risk and tailoring personalized treatment plans for patients at risk of severe PTBE post-surgery. The model demonstrated high predictive accuracy, with AUC values of 0.897 (95% CI: 0.829–0.965) for the training group and 0.719 (95% CI: 0.563–0.876) for the testing group. Additionally, DCA showed substantial net clinical benefit for both training and validation cohorts.

Meningiomas are the most common non-glial brain tumors, accounting for approximately 14–19% of all intracranial lesions (18). Their incidence increases with age, particularly after 65 years (19). Older patients undergoing surgery for these benign tumors are at a significant risk of severe postoperative PTBE, which can lead to adverse outcomes. This condition has been described in various ways, such as “postoperative hemorrhagic infarction,” “venous edema,” or “brain swelling syndrome” related to meningioma (5). In our study, the most severe postoperative PTBE was found to be approximately 11 times larger in volume compared to the tumor itself.

Previous research has indicated that venous infarction can be a complication of meningioma surgery, with occurrence rates reported between 2.0 and 4.0% (20, 21). Convexity and parasagittal meningiomas are especially prone to this due to the complex midline venous structure and the damage to seemingly minor cortical veins (20, 21). However, our investigation identified a post-surgical incidence of vein occlusion or damage at approximately 3.97%, which does not appear to significantly contribute to severe postoperative PTBE.

Although meningiomas primarily occur in extra-axial locations and exhibit slow growth, they can expand significantly, leading to PTBE and hydrocephalus, which pose severe risks of disability and life-threatening complications (22). PTBE is observed in approximately 50–67% of meningioma patients (23, 24), with the edema sometimes exceeding the tumor’s size by two to three times (25). Our study supported these observations, finding a correlation between moderate preoperative PTBE (EI = 1.0–2.0) and an increased likelihood of postoperative PTBE, suggesting a non-linear relationship between preoperative and postoperative PTBE (3). The etiology of PTBE in meningiomas has been extensively studied since the 1980s (7). Research has explored various clinical and pathological factors such as gender, age, tumor location, size, histological subtypes, and venous obstruction. However, these studies have not identified statistically significant or conclusive associations (10). Four primary theories have been proposed to explain the occurrence of PTBE (7). Previous research has suggested that tumor size may play a crucial role in the development of PTBE through brain parenchyma compression theory (26). The Brain Parenchyma Compression Theory suggests that large meningiomas compress the brain, leading to ischemia and cytotoxic edema (27, 28). Recent studies, however, have not consistently demonstrated a significant relationship between tumor size and edema formation, even considering age-related brain atrophy (29–31). Post-resection, severe PTBE can persist or worsen, indicating that tumor size alone may not be a definitive factor. The Secretory-Excretory Theory posits that certain histological subtypes of meningiomas (32) secrete eosinophilic and periodic acid-Schiff (PAS) positive inclusions into perivascular spaces, contributing to PTBE through osmotic mechanisms. Given that these subtypes account for less than 3% of all meningiomas (33), and our study did not include such cases, it is unlikely that this subtype significantly contributes to postoperative PTBE. The Venous Compression Theory suggests that tumors obstructing veins and sinuses can impair blood flow, potentially exacerbating PTBE (7). In our study, MRV scans did not definitively prove venous obstruction as a major contributing factor to PTBE. The Hydrodynamic Theory posits that when the blood supply to a meningioma is insufficient, it secretes angiogenic factors leading to the formation of immature and highly permeable neovessels within the tumor (34, 35). These vessels leak plasma proteins into the tumor. If there is permeability at the tumor-brain interface (absence of an intact arachnoid barrier), these factors diffuse into surrounding brain tissue, promoting vasogenic edema (36). The similarities between PTBE and experimentally induced vasogenic edema support this theory. Hypoplastic efferent tumoral veins (37) may also play a crucial role, allowing diffusion of edema-inducing factors such as vascular endothelial growth factor (VEGF)-A, endothelin-1, and caveolin-1 into adjacent brain tissue (38). This may contribute to both preoperative and postoperative PTBE, particularly in areas adjacent to white matter (14). In summary, while each theory provides insight into the mechanisms of PTBE, the hydrodynamic theory and the role of efferent tumoral veins appear to offer the most comprehensive explanation for the observed edema in meningioma cases.

Previous studies have indicated that the presence of edema in the surrounding brain tissue may suggest compromised integrity of the blood–brain barrier (BBB) and the arachnoid plane, which act as protective barriers against edema formation. While the pia mater allows water and electrolyte permeability, it restricts the passage of plasma proteins. Similarly, the impermeability of the cerebral cortex contributes to maintaining BBB function. Disruption of leptomeningeal and cortical layers can increase vascular permeability, resulting in edema by allowing the influx of plasma proteins and water into white matter (24). Given that meningiomas are typically well-defined tumors located outside brain tissue, they often exhibit a distinct boundary composed of connective tissues such as the pia-arachnoid mater and tumor stroma. This boundary potentially limits the influence that intra-tumoral factors (e.g., VEGF or MMP) may exert on adjacent peri-tumoral brain regions. Therefore, investigating this interface and identifying potential disruptors are crucial for understanding PTBE pathogenesis (39). An anatomical investigation revealed an increased number of vessels crossing the brain-tumor interface in meningiomas associated with preoperative PTBE (39). Surgeons may use bipolar diathermy more frequently at this interface to mitigate post-surgical rebleeding risks due to its higher vascularity. Repositioning retractors during surgery and using bipolar diathermy can potentially induce mechanical trauma and thermal injury, respectively, to the surrounding brain tissue. These factors further compromise blood flow within the already impaired BBB (5). Damage to the surgical cleavage plane between the tumor and cerebral cortex may also result in venous infarction after surgery (40), leading to an enlargement of postoperative PTBE. Previous studies have demonstrated a significant association between the absence of peritumoral rims and a higher risk of PTBE (26, 41, 42). Moreover, having multiple arachnoid layers could potentially mitigate the occurrence of PTBE (7). Our study observed that regions with increased blood supply were more susceptible to medical injury at the brain-tumor interface, which likely played a crucial role in the development of severe PTBE following surgery.

Meningiomas exhibit a high degree of vascularity and blood flow (34). The presence of microvessels with increased permeability within the tumor facilitates surgical and chemotherapy management (43). Nearly all meningiomas show the characteristic “tail of mouse sign,” indicating enhanced meningeal permeability. The vascular properties of meningiomas have been associated with PTBE (44). Although tumor neovascularization is not evident in the edematous region, the hydrostatic pressure exerted by circulating fluid surpasses that of interstitial fluid, resulting in extracellular edema caused by water leakage. Several studies suggest that the increased water content in these tumors may contribute to their growth (41). Recent research has also indicated that cortical blood stealing could play a role. Investigations have revealed the presence of pial arterial blood supply in meningiomas and observed how blood supply from cortical arteries can divert blood from surrounding areas near the tumor (45, 46). This diversion can result in ischemia in the brain tissue surrounding the tumor, leading to PTBE. The dysfunction of the BBB and heightened permeability of capillaries within brain tissue contribute to plasma components and water overflowing into the vicinity of the tumor, thereby causing PTBE with vasogenic origins (45). Significant leakage of contrast agent from the tumor into peritumoral brain tissue is only evident when meningiomas are accompanied by surrounding edema (47). While biopsy can provide insights into the characteristics of tight endothelial junctions and facilitate the evaluation of the microvascular area through immunohistological examination, it requires an invasive procedure (29–31). Our objective was to establish a correlation between the pathology of vascularized meningiomas and post-surgical PTBE, although it could not be considered the primary risk factor.

However, unlike high-grade gliomas, meningiomas typically do not compromise the integrity of BBB unless there is direct brain invasion (26). In meningiomas, a physical barrier often exists between the tumor and the surrounding edematous brain. This observation was consistent with current theories on tumor invasiveness. We also considered factors such as microvascular permeability (48, 49), irregular tumor margins attributed to cortical tumor penetration, tumors with prominent nucleoli producing growth factors like VEGF (9), and a high Ki67 LI indicating active tumor proliferation (34, 50). Our findings were consistent with previous research suggesting that the resolution of PTBE after surgery was not significantly influenced by the proliferative characteristics or invasive behavior of intracranial meningiomas (28).

Our findings suggest that post-surgery PTBE might be associated with BBB disruption and increased tumor water content rather than tumor vascularity. However, this factor provided only an approximate estimate of tumor vascularity, and further research would be needed for additional validation. Previous hypotheses proposed that edema originated from the tumor periphery and propagated through loosely interconnected fibers in the white matter. The higher water content within the tumor might facilitate enhanced fluid diffusion into adjacent brain structures due to pressure gradients, contributing to the development of PTBE (41). However, there has been currently insufficient understanding regarding the relationship between PTBE and surrounding white matter.

Our study faced limitations due to a restricted sample size, necessitating larger sample sizes to prevent overfitting models and ensure consistent results when examining the impact of these prognostic factors. The retrospective nature of our study and its single-center design with a small sample size. In the same time, we faced the post operative CT is not the ideal for estimate the PTBE. Due to the national conditions, in China, most of the patients are poor, and we will try to reduce the treatment costs of patients. Although we hope to improve postoperative examinations such as MRI, we often have to do more than CT to assess the changes in the condition. Although MRI was performed in patients with aggravated PTBE, CT was chosen as the postoperative method in our predictive study. Last but not least, we observed the treatment of steroid and mannitol did not control the PTBE, more studies should do in dealing with this complication.

## Conclusion

Our innovative nomogram, which integrated clinical characteristics, radiological features, and pathological findings, significantly enhanced the accuracy in predicting severe PTBE following meningioma resection. This pioneering tool demonstrated substantial potential in assisting healthcare professionals in developing precise and personalized treatment strategies by providing reliable forecasts regarding severe PTBE after meningioma resection.

## Data Availability

The original contributions presented in the study are included in the article/Supplementary material, further inquiries can be directed to the corresponding author.

## References

[1] Ostrom QT Cioffi G Waite K Kruchko C Barnholtz-Sloan JS. CBTRUS statistical report: primary brain and other central nervous system tumors diagnosed in the United States in 2014-2018. Neuro-Oncology. (2021) 23:iii1–iii105. doi: 10.1093/neuonc/noab200, PMID: 34608945 PMC8491279

[2] Cao J Yan W Li G Zhan Z Hong X Yan H. Incidence and survival of benign, borderline, and malignant meningioma patients in the United States from 2004 to 2018. Int J Cancer. (2022) 151:1874–88. doi: 10.1002/ijc.34198, PMID: 35779059

[3] Li LM Zheng WJ Chen YZ Hu ZH Liao W Lin QC. Predictive factors of postoperative peritumoral brain edema after meningioma resection. Neurol India. (2021) 69:1682–7. doi: 10.4103/0028-3886.333500, PMID: 34979669

[4] Bruno-Mascarenhas MA Ramesh VG Venkatraman S Mahendran JV Sundaram S. Microsurgical anatomy of the superior sagittal sinus and draining veins. Neurol India. (2017) 65:794–800. doi: 10.4103/neuroindia.NI_644_16, PMID: 28681754

[5] Saffarian A Derakhshan N Taghipour M Eghbal K Rouhi A. "Wounded meningioma syndrome": postoperative exacerbation of brain edema in brain-invasive meningioma. World Neurosurg. (2018) 115:483–4. doi: 10.1016/j.wneu.2018.03.106, PMID: 29958375

[6] Fukamachi A Koizumi H Nukui H. Postoperative intracerebral hemorrhages: a survey of computed tomographic findings after 1074 intracranial operations. Surg Neurol. (1985) 23:575–80. doi: 10.1016/0090-3019(85)90006-0, PMID: 3992457

[7] Berhouma M Jacquesson T Jouanneau E Cotton F. Pathogenesis of peri-tumoral edema in intracranial meningiomas. Neurosurg Rev. (2019) 42:59–71. doi: 10.1007/s10143-017-0897-x, PMID: 28840371

[8] Vignes JR Sesay M Rezajooi K Gimbert E Liguoro D. Peritumoral edema and prognosis in intracranial meningioma surgery. J Clin Neurosci. (2008) 15:764–8. doi: 10.1016/j.jocn.2007.06.001 18406142

[9] Bebawy JF. Perioperative steroids for peritumoral intracranial edema: a review of mechanisms, efficacy, and side effects. J Neurosurg Anesthesiol. (2012) 24:173–7. doi: 10.1097/ANA.0b013e3182578bb5, PMID: 22544067

[10] Tamiya T Ono Y Matsumoto K Ohmoto T. Peritumoral brain edema in intracranial meningiomas: effects of radiological and histological factors. Neurosurgery. (2001) 49:1046–51. doi: 10.1227/00006123-200111000-00003, PMID: 11846896

[11] Chen Q Zhang Y Zhang M Li Z Liu J. Application of machine learning algorithms to predict acute kidney injury in elderly orthopedic postoperative patients. Clin Interv Aging. (2022) 17:317–30. doi: 10.2147/CIA.S349978, PMID: 35386749 PMC8979591

[12] Gao L Chang Y Lu S Liu X Yao X Zhang W. A nomogram for predicting the necessity of tracheostomy after severe acute brain injury in patients within the neurosurgery intensive care unit: a retrospective cohort study. Heliyon. (2024) 10:e27416. doi: 10.1016/j.heliyon.2024.e27416, PMID: 38509924 PMC10951500

[13] Rao D Yang L Enxi X Siyuan L Yu Q Zheng L. A predictive model in patients with chronic hydrocephalus following aneurysmal subarachnoid hemorrhage: a retrospective cohort study. Front Neurol. (2024) 15:1366306. doi: 10.3389/fneur.2024.1366306, PMID: 38817542 PMC11137279

[14] Osawa T Tosaka M Nagaishi M Yoshimoto Y. Factors affecting peritumoral brain edema in meningioma: special histological subtypes with prominently extensive edema. J Neuro-Oncol. (2013) 111:49–57. doi: 10.1007/s11060-012-0989-y, PMID: 23104516

[15] Wang C Zhao Y Jin B Gan X Liang B Xiang Y. Development and validation of a predictive model for coronary artery disease using machine learning. Front Cardiovasc Med. (2021) 8:614204. doi: 10.3389/fcvm.2021.614204, PMID: 33634169 PMC7902072

[16] Hou N Li M He L Xie B Wang L Zhang R. Predicting 30-days mortality for MIMIC-III patients with sepsis-3: a machine learning approach using XGboost. J Transl Med. (2020) 18:462. doi: 10.1186/s12967-020-02620-5, PMID: 33287854 PMC7720497

[17] Ngiam KY Khor IW. Big data and machine learning algorithms for health-care delivery. Lancet Oncol. (2019) 20:e262–73. doi: 10.1016/S1470-2045(19)30149-4, PMID: 31044724

[18] Ketter R Rahnenführer J Henn W Kim YJ Feiden W Steudel WI. Correspondence of tumor localization with tumor recurrence and cytogenetic progression in meningiomas. Neurosurgery. (2008) 62:61–9. doi: 10.1227/01.NEU.0000311062.72626.D6, PMID: 18300892

[19] Kshettry VR Ostrom QT Kruchko C Al-Mefty O Barnett GH Barnholtz-Sloan JS. Descriptive epidemiology of World Health Organization grades II and III intracranial meningiomas in the United States. Neuro-Oncology. (2015) 17:1166–73. doi: 10.1093/neuonc/nov069, PMID: 26008603 PMC4490879

[20] Sughrue ME Rutkowski MJ Shangari G Fang S Parsa AT Berger MS. Incidence, risk factors, and outcome of venous infarction after meningioma surgery in 705 patients. J Clin Neurosci. (2011) 18:628–32. doi: 10.1016/j.jocn.2010.10.001, PMID: 21349725

[21] Giordan E Sorenson TJ Lanzino G. Optimal surgical strategy for meningiomas involving the superior sagittal sinus: a systematic review. Neurosurg Rev. (2020) 43:525–35. doi: 10.1007/s10143-018-1026-1 30171502

[22] Marosi C Hassler M Roessler K Reni M Sant M Mazza E. Meningioma. Crit Rev Oncol Hematol. (2008) 67:153–71. doi: 10.1016/j.critrevonc.2008.01.010 18342535

[23] Kim BW Kim MS Kim SW Chang CH Kim OL. Peritumoral brain edema in meningiomas: correlation of radiologic and pathologic features. J Korean Neurosurg Soc. (2011) 49:26–30. doi: 10.3340/jkns.2011.49.1.26, PMID: 21494359 PMC3070891

[24] Hou J Kshettry VR Selman WR Bambakidis NC. Peritumoral brain edema in intracranial meningiomas: the emergence of vascular endothelial growth factor-directed therapy. Neurosurg Focus. (2013) 35:E2. doi: 10.3171/2013.8.FOCUS13301, PMID: 24289127

[25] Mattei TA Mattei JA Ramina R Aguiar PH Plese JP Marino R Jr. Edema and malignancy in meningiomas. Clinics (Sao Paulo). (2005) 60:201–6. doi: 10.1590/S1807-59322005000300004, PMID: 15962080

[26] Sapkota MR Yang Z Zhu D Zhang Y Yuan T Gao J. Evaluation of epidemiologic factors, radiographic features, and pathologic findings for predicting peritumoral brain edema in meningiomas. J Magn Reson Imaging. (2020) 52:174–82. doi: 10.1002/jmri.27046, PMID: 31922353

[27] Huang Q Zhao SL Tian XY Li B Li Z. Increased co-expression of macrophage migration inhibitory factor and matrix metalloproteinase 9 is associated with tumor recurrence of meningioma. Int J Med Sci. (2013) 10:276–85. doi: 10.7150/ijms.5185, PMID: 23372434 PMC3558716

[28] Paek SH Kim CY Kim YY Park IA Kim MS Kim DG. Correlation of clinical and biological parameters with peritumoral edema in meningioma. J Neuro-Oncol. (2002) 60:235–45. doi: 10.1023/A:1021186401522 12510775

[29] Yin T Zhang J Zhang H Zhao Q Wei L Wang S. Poor brain-tumor Interface-related edema generation and cerebral venous decompensation in parasagittal meningiomas. World Neurosurg. (2018) 115:e544–51. doi: 10.1016/j.wneu.2018.04.092, PMID: 29689390

[30] Lee KJ Joo WI Rha HK Park HK Chough JK Hong YK. Peritumoral brain edema in meningiomas: correlations between magnetic resonance imaging, angiography, and pathology. Surg Neurol. (2008) 69:350–5. doi: 10.1016/j.surneu.2007.03.027, PMID: 18262249

[31] Inamura T Nishio S Takeshita I Fujiwara S Fukui M. Peritumoral brain edema in meningiomas–influence of vascular supply on its development. Neurosurgery. (1992) 31:179–85. doi: 10.1227/00006123-199208000-00002, PMID: 1513424

[32] Regelsberger J Hagel C Emami P Ries T Heese O Westphal M. Secretory meningiomas: a benign subgroup causing life-threatening complications. Neuro-Oncology. (2009) 11:819–24. doi: 10.1215/15228517-2008-109, PMID: 19066343 PMC2802401

[33] Wang DJ Xie Q Gong Y Wang Y Cheng HX Mao Y. Secretory meningiomas: clinical, radiological and pathological findings in 70 consecutive cases at one institution. Int J Clin Exp Pathol. (2013) 6:358–74. PMID: 23412548 PMC3563189

[34] Nassehi D. Intracranial meningiomas, the VEGF-A pathway, and peritumoral brain oedema. Dan Med J. (2013) 60:B4626. PMID: 23651727

[35] Nassehi D Dyrbye H Andresen M Thomsen C Juhler M Laursen H. Vascular endothelial growth factor a protein level and gene expression in intracranial meningiomas with brain edema. APMIS. (2011) 119:831–43. doi: 10.1111/j.1600-0463.2011.02764.x 22085359

[36] Ding YS Wang HD Tang K Hu ZG Jin W Yan W. Expression of vascular endothelial growth factor in human meningiomas and peritumoral brain areas. Ann Clin Lab Sci. (2008) 38:344–51. 18988927

[37] Tanaka M Imhof HG Schucknecht B Kollias S Yonekawa Y Valavanis A. Correlation between the efferent venous drainage of the tumor and peritumoral edema in intracranial meningiomas: superselective angiographic analysis of 25 cases. J Neurosurg. (2006) 104:382–8. doi: 10.3171/jns.2006.104.3.382, PMID: 16572650

[38] Gawlitza M Fiedler E Schob S Hoffmann KT Surov A. Peritumoral brain edema in meningiomas depends on Aquaporin-4 expression and not on tumor grade, tumor volume, cell count, or Ki-67 labeling index. Mol Imaging Biol. (2017) 19:298–304. doi: 10.1007/s11307-016-1000-7, PMID: 27552812

[39] Nakasu S Fukami T Jito J Matsuda M. Microscopic anatomy of the brain-meningioma interface. Brain Tumor Pathol. (2005) 22:53–7. doi: 10.1007/s10014-005-0187-0, PMID: 18095106

[40] Jang WY Jung S Jung TY Moon KS Kim IY. Predictive factors related to symptomatic venous infarction after meningioma surgery. Br J Neurosurg. (2012) 26:705–9. doi: 10.3109/02688697.2012.690914, PMID: 22702388

[41] Nakano T Asano K Miura H Itoh S Suzuki S. Meningiomas with brain edema: radiological characteristics on MRI and review of the literature. Clin Imaging. (2002) 26:243–9. doi: 10.1016/S0899-7071(02)00433-3, PMID: 12140153

[42] Fiani B Jarrah R Bhandarkar AR de Stefano F Amare A Aljameey UA. Peritumoral edema in meningiomas: pathophysiology, predictors, and principles for treatment. Clin Transl Oncol. (2023) 25:866–72. doi: 10.1007/s12094-022-03009-0, PMID: 36427121

[43] Hussain NS Moisi MD Keogh B McCullough BJ Rostad S Newell D. Dynamic susceptibility contrast and dynamic contrast-enhanced MRI characteristics to distinguish microcystic meningiomas from traditional grade I meningiomas and high-grade gliomas. J Neurosurg. (2017) 126:1220–6. doi: 10.3171/2016.3.JNS14243, PMID: 27285539

[44] Bitzer M Wöckel L Luft AR Wakhloo AK Petersen D Opitz H. The importance of pial blood supply to the development of peritumoral brain edema in meningiomas. J Neurosurg. (1997) 87:368–73. doi: 10.3171/jns.1997.87.3.0368, PMID: 9285600

[45] Xiang L Sun LH Liu B Wang LS Gong XJ Qiu J. Quantitative dynamic contrast-enhanced magnetic resonance imaging for the analysis of microvascular permeability in peritumor brain edema of fibrous meningiomas. Eur Neurol. (2021) 84:361–7. doi: 10.1159/000516921, PMID: 34315157

[46] Dubel GJ Ahn SH Soares GM. Contemporary endovascular embolotherapy for meningioma. Semin Intervent Radiol. (2013) 30:263–77. doi: 10.1055/s-0033-1353479, PMID: 24436548 PMC3773039

[47] Bitzer M Nägele T Geist-Barth B Klose U Grönewäller E Morgalla M. Role of hydrodynamic processes in the pathogenesis of peritumoral brain edema in meningiomas. J Neurosurg. (2000) 93:594–604. doi: 10.3171/jns.2000.93.4.0594, PMID: 11014537

[48] Dewhirst MW Ashcraft KA. Implications of increase in vascular permeability in tumors by VEGF: a commentary on the pioneering work of Harold Dvorak. Cancer Res. (2016) 76:3118–20. doi: 10.1158/0008-5472.CAN-16-1292, PMID: 27251086

[49] Lugano R Ramachandran M Dimberg A. Tumor angiogenesis: causes, consequences, challenges and opportunities. Cell Mol Life Sci. (2020) 77:1745–70. doi: 10.1007/s00018-019-03351-7, PMID: 31690961 PMC7190605

[50] Bečulić H Skomorac R Jusić A Alić F Mašović A Burazerović E. Correlation of peritumoral brain edema with morphological characteristics and KI67 proliferative index in resected intracranial meningiomas. Acta Clin Croat. (2019) 58:42–9. doi: 10.20471/acc.2019.58.01.06 PMC6629213 31363324

[51] Gordon Y Partovi S Müller-Eschner M Amarteifio E Bäuerle T Weber MA. Dynamic contrast-enhanced magnetic resonance imaging: fundamentals and application to the evaluation of the peripheral perfusion. Cardiovasc Diagn Ther. (2014) 4:147–64. doi: 10.3978/j.issn.2223-3652.2014.03.01, PMID: 24834412 PMC3996240

