# Supplementary figures and images for: Development of a clinical-radiological nomogram for predicting severe postoperative peritumoral brain edema following intracranial meningioma resection
Source: Front Neurol. 2025 Jan 16;15:1478213. doi: 10.3389/fneur.2024.1478213 (PMC11780903; doi:10.3389/fneur.2024.1478213)

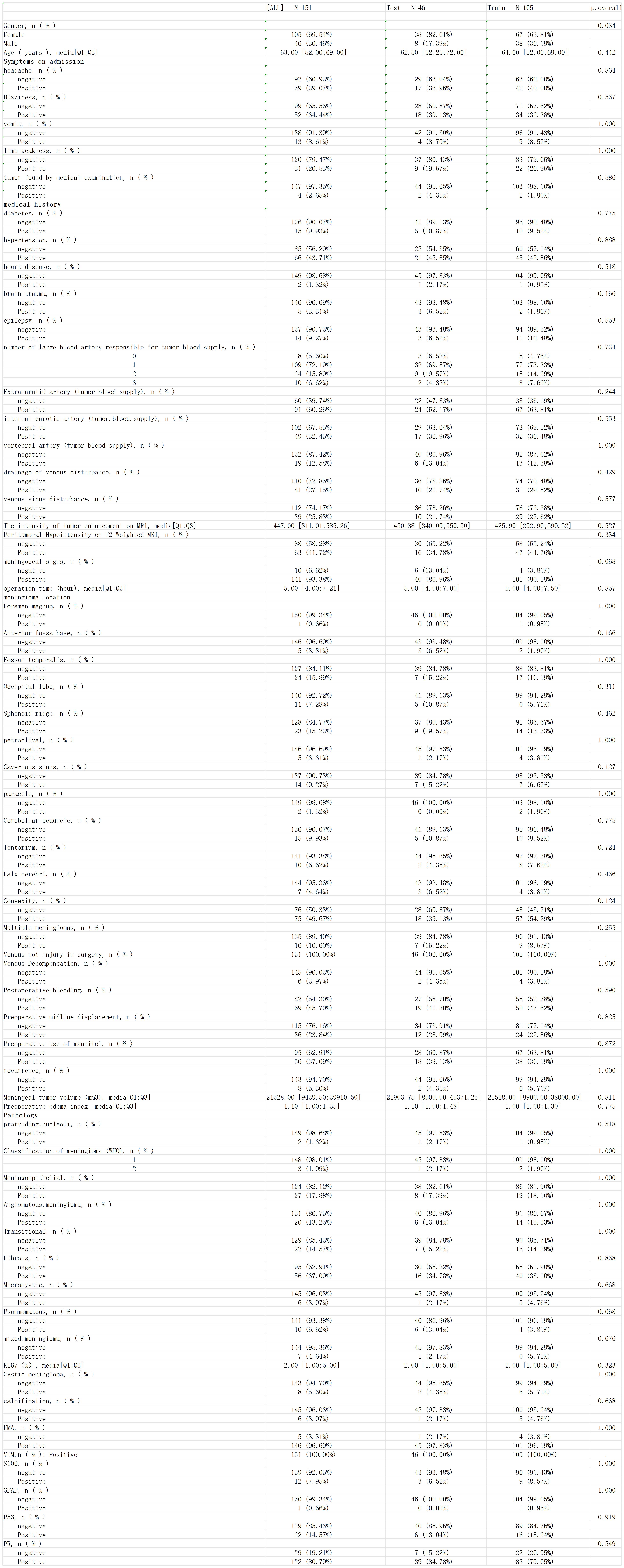

Supplement: Supplementary file 1 [file Image_1.JPEG]

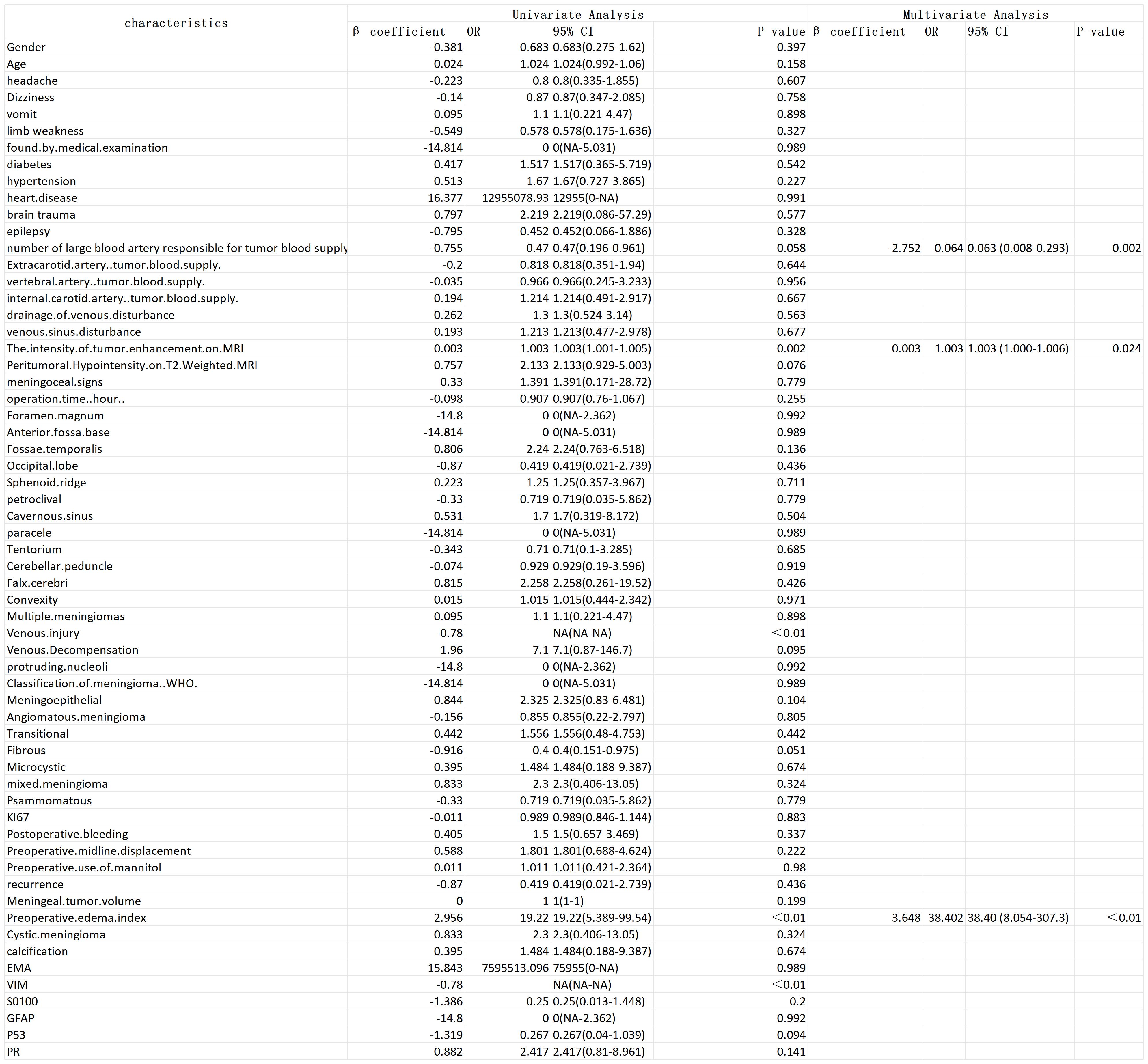

Supplement: Supplementary file 2 [file Image_2.JPEG]
